# Serological evidence and experimental infection of cynomolgus macaques with pteropine orthoreovirus reveal monkeys as potential hosts for transmission to humans

**DOI:** 10.1080/22221751.2019.1621668

**Published:** 2019-05-28

**Authors:** Chee Wah Tan, Kevin Wittwer, Xiao Fang Lim, Anna Uehara, Shailendra Mani, Lin-Fa Wang, Danielle E. Anderson

**Affiliations:** a Programme in Emerging Infectious Diseases, Duke-NUS Medical School, Singapore; b Veterinary Medicine Division, Paul-Ehrlich-Institute, Langen, Germany

**Keywords:** Pteropine, orthoreovirus, cynomolgus macaque, bat, zoonosis

## Abstract

Pteropine orthoreoviruses (PRV) are emerging bat-borne viruses with proven zoonotic transmission. We recently demonstrated human exposure to PRV in Singapore, which together with previous reports from Malaysia and Vietnam suggest that human infection of PRV may occur periodically in the region. This raises the question whether bats are the only sources of human infection. In this study, we screened 517 cynomolgus macaques caught in Singapore for evidence of exposure to PRV3M (also known as Melaka virus), which was first isolated from human patients in Melaka, Malaysia. We found that 67 serum samples were PRV3M positive by ELISA and 34 were also positive by virus neutralization assay. To investigate whether monkeys could act as hosts for PRV transmission, we experimentally infected cynomolgus macaques with PRV3M and housed these animals with uninfected monkeys. Although no clinical signs of infection were observed in infected animals, viral RNA was detected in nasal and rectal swabs and all infected macaques seroconverted. Additionally, one of the uninfected animals seroconverted, implying active shedding and transmission of PRV3M. We provide evidence that PRV exposure in the macaque population in Singapore occurs at a relatively high prevalence and this study suggests that cynomolgus macaques may be an intermediate or reservoir host for PRVs.

## Introduction

Pteropine orthoreoviruses (PRVs) are a group of emerging bat-borne viruses, belonging to the genus *Orthoreovirus* within the family *Reoviridae*. PRV virions are non-enveloped, fusogenic, and contain double-stranded RNA genomes with ten segments (S1, S2, S3, S4, M1, M2, M3, L1, L2 and L3).

PRV1N (alternatively known as Nelson Bay virus) was first isolated in 1968 from a grey-headed flying fox (*Pteropus policephalus*) in Nelson Bay, Australia [1]. The second PRV isolated was PRV2P (alternatively known as Pulau virus) in 1999 from pooled urine samples from a fruit bat (*Pteropus hypomelanus*) in Tioman Island, Malaysia [2]. In 2006, PRV3M (alternatively known as Melaka virus) was isolated during the investigation of an outbreak of a severe respiratory and enteric disease among different members of a family in Melaka, Malaysia [3]. PRV infections in humans vary from asymptomatic or mild flu-like to severe respiratory diseases. Conversely, in an experimental infection, nine-week old female Balb/c mice intranasally infected with of 1 × 10^6^ of PRV-MB (PRV10M, alternatively known as Miyazaki-Bali /2007 [4]) developed clinical signs of disease (piloerection, slowness in movement, anorexia and weight loss) from 2 days post-infection and all mice died by 6 days post-infection [5].

**Figure 1.**
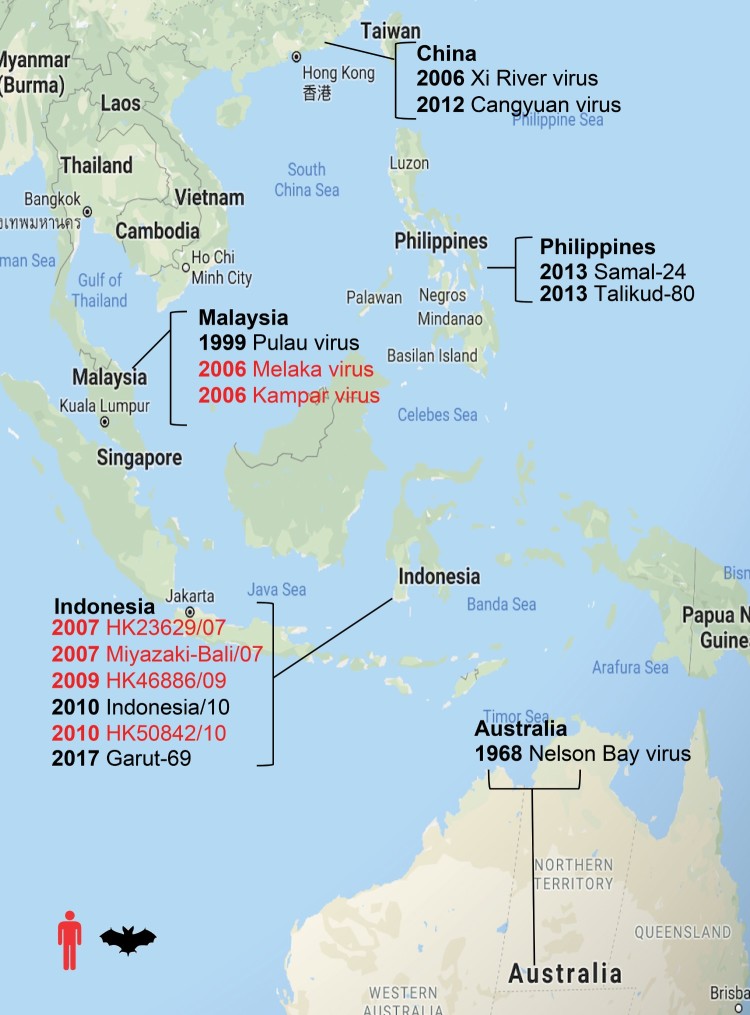
Map of representative PRV detections. Year of PRV outbreaks or isolation noted by country. Isolations from humans are shown in red and isolations from bats are shown in black.

Appreciation of both the diversity and the zoonotic potential of PRVs increased following the isolation of PRV4K (alternatively known as Kampar virus), yet another PRV isolated from humans with acute respiratory illness in Kampar, Malaysia [6]. Evidence of PRV infection in humans has been reported in various locations in Southeast Asia (Figure 1). In a molecular surveillance study in Rembau, Malaysia, PRV was detected in oropharyngeal swabs from 17% of out-patients suffering from acute respiratory tract infections [7] and in serological studies on select populations, PRV seroprevalence rates range from 4.4–13% [7–9]. Although direct bat-to-human transmission was likely the route of transmission in the PRV3M outbreak [3], it is less clear whether bats played a direct role in transmitting the viruses to humans in the other reported cases of human infection. The high prevalence in selected populations from the recent surveillance studies supports the possibility of transmission by reservoir or intermediate hosts, which may have closer contact with humans than bats.

To date, there have been at least 15 different accounts identifying PRVs from either human outbreak investigations or bat virome studies [1–4,6,10–17]. It is well known that interspecies transmission of bat-borne viruses is a source of disease outbreaks in the human population and indeed evidence of bat-to-human transmission of PRV has been demonstrated [3,13]. In contrast to all previous discoveries, non-human primates were the source of the most recent PRV detection. Given the cross-species transmission of PRVs and frequent human-monkey interaction in Singapore and surrounding regions, we initiated this study to determine the seroprevalence of PRV3M in wild cynomolgus macaques and to examine the susceptibility of cynomolgus macaques to experimental infection with PRV3M and their potential role in acting as hosts facilitating cross-species transmission. Non-human primates may serve as natural reservoirs and maintain PRV, or act as intermediate hosts to provide a link for virus transmission between bats and humans.

During the preparation of this manuscript, the isolation and characterization of an orthoreovirus from monkey faeces collected in Lopburi Province, Thailand was reported [18]. This supports our original hypothesis that animals other than bats may be susceptible to PRV infection and in turn play a role for cross-species transmission.

## Materials and methods

### Cells and viruses

Vero cells (African green monkey, ATCC#CCL-81) cells were maintained in Dulbecco’s modified Eagle medium (DMEM, Invitrogen) supplemented with 10% foetal bovine serum (FBS, Invitrogen) at 37°C and 5% CO_2_. PRV3M (gift from Kaw Bing Chua from Temasek Lifescience Laboratory, Singapore) was amplified in Vero cells. Upon 80-90% virus-induced cytopathic effect (CPE), viruses were harvested, clarified by centrifugation at 4,000 x *g* for 10 min and the virus-containing supernatant was stored in −80°C. Titres were determined by limiting dilution. In brief, 10-fold serial diluted virus was added into a 96-well plate containing 1 × 10^4^ Vero cells per well. Cells were observed for CPE for 5 days post-infection and the titres were expressed as TCID_50_/ml.

### Purification of PRV3M

Clarified PRV3M virus-containing supernatant was overlaid on 2 ml of 20% sucrose in an ultra-clear centrifuge tube (Beckman coulter) and was centrifuged at 125,000 × g for 90 min (SW41 rotor in Opitma XPN-100 ultracentrifuge). After centrifugation, the supernatant was gently removed and the pellet was resuspended with DPBS (Invitrogen) and stored in −80°C.

### PRV3M ELISA

To establish a PRV ELISA based on purified whole-virus antigen, 100 μl of purified PRV3M was coated on MAXISORP Nunc Immuno plates (Nunc, USA) in carbonate coating buffer (0.1 M NaHCO_3_, 0.1 M Na_2_HCO_3_, pH 9.6) overnight at 4°C. Following coating, plates were blocked with 100 μL of OptEIA (BD Bioscience, USA) for 1 h at room temperature. Each monkey serum sample was diluted to 1:200 in OptEIA and 50 μL was added to the well and incubated for 1 h. Wells were washed 5 times with 200 μL of PBST, then 50 μL of HRP-conjugated anti-monkey secondary antibody (Santa-Cruz, USA) was added at a 1:10,000 dilution for 1 h. Wells were washed 5 times and 50 μL TMB substrate (Life technologies) was added and incubated at room temperature for 15 min. Following incubation, 50 μL of stop solution (KPL) was added and absorbance readings were measured at 450 nm using a Cytation 5 microplate reader (BioTek, USA).

### Serum neutralization test

Serum neutralization tests were performed by incubating 200 TCID_50_ of PRV3M with 2-fold serial diluted macaque serum for 30 min at 37°C. The virus-antibody mixture was then added to a monolayer of Vero cells and incubated for 1 h at 37°C. After incubation, the inoculum was removed and replaced with DMEM supplemented with 2% FBS. Virus-induced CPE was observed at days 4 post-infection and the titre was recorded at the highest dilution where CPE was absent.

### PRV3M Western blot

6 × 10^5^ TCID_50_ of purified PRV3M was resolved by SDS-PAGE on a 12% gel. The proteins were transferred onto a PVDF membrane, and blocked for 1 h in 5% BSA in Tris-buffered saline containing 0.05% Tween-20 (TBS-T). Membranes were incubated with monkey sera diluted to 1:500 in 5% BSA in TBS-T for 1 h at room temperature. Membranes were washed 3 times in TBS-T, then HRP-conjugated anti-monkey antibody (Santa-Cruz, USA) at a 1:10,000 dilution was added for 1 h. The immuno-signal was developed using Amersham ECL western blotting detection reagent (GE healthcare, UK) and images were captured using ChemiDoc MP imaging system (Bio-Rad, USA).

### Animal experiments

Cynomolgus macaques (*Macaca fascicularis*) were used for all studies. The experiments were approved by the SingHealth Institutional Animal Care and Use Committee of the SingHealth Experimental Medicine Centre (SEMC IACUC Reference: 2017/SHS/1333). Cynomolgus macaques were purchased from the SingHealth colony and free of antibodies against PRV3M as determined by VNT and ELISA. In a pilot study, three animals were infected via the intranasal route with 1 × 10^8^ TCID_50_ of PRV3M and three animals were uninfected. All animals were housed in the same room in individual cages. The cages were placed next to each other, so the animals could have physical contact through the cages. Due to the nature of the open cages system, virus transmission could either be through contact or aerosol. Animals were observed daily for activity and clinical signs. The animals were anaesthetized with 10–15 mg/kg Ketamine and blood samples were collected from the femoral vein on days 1–7, 9, 12, 14, 21, 28, 35 and 42. Each time the animals were anaesthetized, body temperature was measured rectally, weight recorded and oral and rectal swabs collected. Chest X-rays were taken on -2, 7 and 21 days post-infection using a Fuji-film digital X-Ray. Powered Air Purifying Respirators were worn by all personnel while handling monkeys.

### RNA purification and in vitro transcription

RNA was isolated from monkey serum samples using ZR Viral RNA (Zymo Research) according to the manufacturer’s instructions. RNA was isolated from oral and rectal swabs using E.Z.N.A. Total RNA Kit I (Omega Biotek) according to the manufacturer’s instructions. Templates for *in vitro* transcription were generated by digesting a plasmid containing a T7 promotor and the PRV3M S4 gene with *Bam*HI. The digested plasmid was reverse transcribed using HiScribe T7 High Yield RNA Synthesis Kit (NEB). DNA was removed by DNase treatment with DNase I (NEB) for 15 min at 37°C and the RNA purified using RNeasy mini kit (Qiagen). The total amount of RNA was quantified using a Nanodrop (ThermoScientific). The number of RNA molecules produced by in vitro transcription was calculated using the following formula: RNA molecules = (Amount of RNA in ng × 6.0221 × 10^23^ molecules/mole)/ (Length × 340 g/mole × 10^9 ^ng/g).

### Quantitative real-time PCR

Quantitative PCR (qPCR) was performed using QuantiTect Probe RT–PCR Kit (Qiagen) reagents and with the CFX96 Real-Time System (Bio-Rad). Each 25 µL PCR reaction contained 12.5 µL 2X QuantiTect PCR master mix, 1 µL of each 10 mM primer, 0.5 μL 0.2 mM probe, 0.5 μL reverse transcriptase, 3 µL RNA template and 6.5 µL H_2_O. Every PCR was performed as follows: reverse transcription at 50°C for 10 min, initial PCR activation at 95°C for 5 min and 45 amplification cycles consisting of a 95°C denaturation for 10 sec and a 60°C annealing/extension for 30 s. Sequences of primers and probes are as follows; PRV3M-S4-F: 5′-CAT TGT CAC TCC GAT TAT GG -3′, PRV3M-S4-R; 5′- TGG GAG GGT GCA GAG CAT AG -3′, PRV3M-S4-probe5′- /56-FAM/ GTA GGC ATG CCG CTC GTG GAA TCC A /3BHQ_1/ -3′. Each PCR was performed in duplicate to obtain an average Ct for each sample. Amplicons were quantified by plotting the Ct values against standard curves made using 10-fold dilutions of cDNA produced from *in vitro* transcription RNA samples. Data were expressed as molecules of RNA.

## Results

### Serological evidence indicates PRV is circulating in wild cynomolgus macaques in Singapore

**Figure 2.**
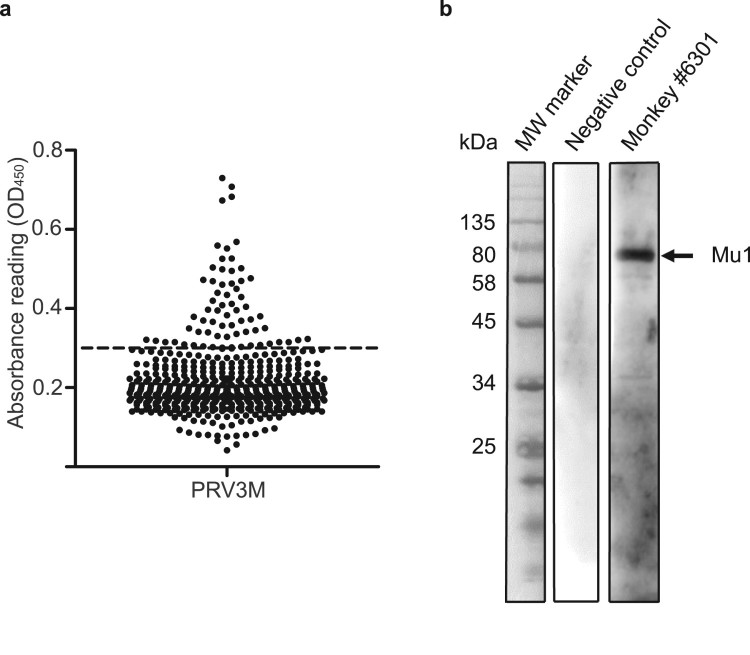
Serological analysis of macaque sera on PRV3M. (a) Whole virus ELISA of macaque sera. PRV3M particles were purified by ultracentrifugation and were used as the antigen for ELISA analysis. Cut-off value was determined by 3 standard deviation of the geometric mean. (b) Determination of the immune-dominant epitopes of PRV3M. Purified PRV3M particles were used as the antigen for Western blot analysis. Serum from monkey #6301 was used to detect PRV3M antigens. PRV3M Mu1 is indicated with an arrow.

A total of 517 serum samples from wild-caught cynomolgus macaques were collected from 2010 to 2017 in Singapore. An initial screening was performed using a PRV3M whole virus-based ELISA. By using three standard deviations of the geometric mean as a cut-off, 67 serum samples were identified as reactive to PRV3M (Figure 2(a), Table 1). To further confirm the positivity of the samples and increase specificity, a serum neutralization test against PRV3M was performed on all ELISA positive serum samples. A total of 34 serum samples were able to neutralize PRV3M at a dilution of at least 1:10 (Table 1). To further confirm the specificity, Western blot analysis was conducted using purified virions. Shown in Figure 2(b) is a representative Western blot conducted with sample #6301, a known positive sample with a high neutralizing antibody titre, and a negative control sample, both used at dilution of 1:500. The Western blot analysis not only confirmed findings from ELISA and VNT, but also revealed that the major outer capsid protein (mu1) was the most reactive viral protein (Figure 2(b)).

**Table 1.** PRV3M seropositivity in cynomolgus macaques.

No.	Monkey ID	Year of capture	ELISA^a^	Neutralization
1	9	2010	+	80
2	36	2011	+	<10
3	37	2011	+	<10
4	39	2011	++	<10
5	63	2011	+	<10
6	64	2011	+	<10
7	80	2012	+	<10
8	82	2012	+	<10
9	84	2012	+	10
10	85	2012	++	40
11	86	2012	+++	<10
12	87	2012	++	160
13	89	2012	++	<10
14	91	2012	++	160
15	96	2013	++	160
16	196	2013	+	<10
17	260	2013	+++	≥320
18	328	2013	+	160
19	475	2013	++	160
20	492	2013	++	160
21	511	2013	+	<10
22	528	2013	++	≥320
23	653	2014	+	<10
24	666	2014	+	80
25	683	2014	+	40
26	684	2014	+++	≥320
27	698	2014	+	<10
28	706	2014	+	<10
29	721	2014	++	80
30	734	2014	++	160
31	740	2014	+	<10
32	763	2014	++	160
33	765	2014	+	<10
34	786	2014	++	160
35	801	2014	+	<10
36	822	2014	+++	320
37	838	2014	+++	320
38	864	2014	++	20
39	893	2014	+	40
40	920	2015	+++	≥320
41	962	2015	+	<10
42	1028	2015	+	80
43	1111	2015	+	<10
44	1163	2015	+	<10
45	1167	2015	+	<10
46	1204	2015	+++	≥320
47	1211	2015	+	<10
48	1240	2015	+	<10
49	1258	2015	+++	160
50	1302	2015	+	<10
51	1330	2015	+	<10
52	1338	2015	++	160
53	1358	2015	+	<10
54	1392	2015	+	<10
55	1401	2015	+	<10
56	1404	2015	+	<10
57	2933	2016	+	10
58	5207	2016	+	<10
59	5223	2016	+++	80
60	5288	2016	+	<10
61	5332	2016	+	<10
62	5333	2016	+	<10
63	6301	2017	+++	≥320
64	6316	2017	++	20
65	6330	2017	+	<10
66	6719	2017	+++	40
67	8152	2017	+	<10

^a^Absorbance reading (OD_450_) values of 0.30–0.39 (+), 0.4–0.49 (++) and >0.5 (+++).

### PRV3M infection is subclinical in cynomolgus macaques

**Figure 3.**
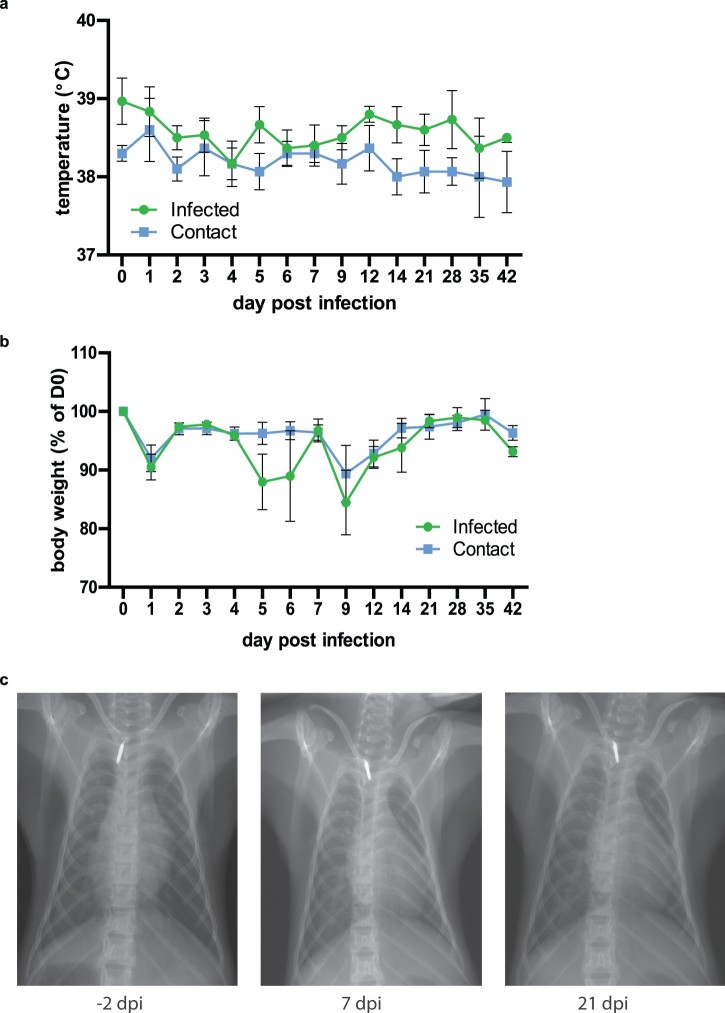
Clinical manifestations in PRV3M-infected cynomolgus macaques. (a) Body temperature of monkeys after intranasal infection with PRV3M (b) Changes in body weight after intranasal infection with PRV3M are displayed as percentage of initial weight. (c) Chest x-ray on -2, 7 and 21 d.p.i.

To assess the clinical disease spectrum of PRV3M, we infected three adult cynomolgus macaques with 1 × 10^8^ TCID_50_ PRV3M via the intranasal route. Only animals seronegative to PRV were used for this study. Blood and swab samples were collected daily for the first 7 days, and then on days 9, 12, 21, 28, 35 and 42 from anaesthetized animals. Body temperature and weight were recorded every time animals were anaesthetized. Infected animals did not experience a significant temperature rise, although the body temperature of the infected group of animals was slightly higher overall than the contact group for the duration of the study (Figure 3(a)). No significant weight loss occurred in either group, but there was a fluctuation in the infected group between days 5–9 post-infection (Figure 3(b)). Chest X-rays were normal in all animals when visualized on days -2, 7 and 21 post-infection (Figure 3(c)). No additional clinical signs such as rash, loss of appetite, diarrhoea, dehydration, depression or inactivity were observed throughout the experimental period.

We investigated the viral kinetics in serum, and oral and rectal swabs. Viremia was not evident in any of the time points measured. Viral RNA was detected in throat swabs of infected animals from day 1 post-infection (Table 2) and was sporadically present until day 6 post-infection. Viral RNA was detected in 2 of the 3 infected monkeys in rectal swabs.

**Table 2.** Detection of PRV3M RNA in oral and rectal swabs from experimentally infected macaques.

Monkey	5239		5244		6309	
Swab	Throat	Rectal	Throat	Rectal	Throat	Rectal
D1	1.74 × 10^6^	2.19 × 10^3^	5.34 × 10^6^	-	1.20 × 10^7^	-
D2	6.54 × 10^5^	–	2.29 × 10^5^	-	4.75 × 10^7^	-
D3	–	2.82 × 10^5^	–	–	1.30 ×10^4^	–
D4	–	1.00 × 10^7^	–	–	3.94 × 10^5^	2.51 × 10^6^
D5	–	–	–	–	3.79 × 10^5^	–
D6	–	7.18 × 10^6^	–	–	–	–
D7	–	2.82 × 10^3^	–	–	–	–
D9	–	–	–	–	–	–
D12	–	–	–	–	–	–
D14	–	–	–	–	–	–
D21	–	–	–	–	–	–

### Transmission of PRV3M occurs between cynomolgus macaques

To evaluate the hypothesis that macaques can serve as an intermediate host for spillover of PRV, we monitored the occurrence of transmission of PRV3M from experimentally infected to naïve monkeys. Similar to the infected animals, none of the naïve animals displayed any clinical signs of disease and viremia was not detected (data not shown). In contrast to the infected animals, viral RNA was not detected on rectal and oral swabs, with the exception of a trace reading on day 3 post-infection in a rectal swab of one animal (monkey #6310) and in the oral swab of another animal at day 5 post-infection (monkey #6308). Virus isolation was not attempted in this pilot study.

To confirm infection in the absence of clinical signs in both infected and contact animals, serum neutralization tests were performed from all monkeys on days 0, 21, 28, 35 and 42 post-infection (Table 3). All 3 infected animals had seroconverted by day 21 post-infection with titres of 80. One monkey from the contact group (monkey #6310) seroconverted by day 21 post-exposure and the neutralization titre increased until day 42, similar to the infected animals. These results provide evidence of PRV3M transmission between cynomolgus macaques.

**Table 3.** PRV3M antibody titres in cynomolgus macaques.

Monkey	Days post-infection				
	0	21	28	35	42
5239 (infected)	<10	80	320	≥640	≥640
5244 (infected)	<10	80	320	320	≥640
6309 (infected)	<10	80	320	≥640	320
5261 (contact)	<10	<10	<10	<10	<10
6308 (contact)	<10	<10	<10	<10	<10
6310 (contact)	<10	40	320	≥640	≥640

## Discussion

Bat-borne zoonotic viruses have been responsible for several important emerging zoonotic infectious disease outbreaks in the last two decades including Hendra virus, Nipah virus, Ebola virus, SARS and MERS coronaviruses [19–24]. It is important to note that, almost in all of the cases, an intermediate host has been identified to play a crucial role in amplifying and transmitting the virus from bats to humans such as horses for Hendra virus, pigs for Nipah virus and palm civets for SARS-CoV [25–29]. With multiple reports of PRV presence in bats in many parts of Asia [2,12,14–17] and a higher than expected prevalence in certain human populations [7–9], we raised the hypothesis that it is possible that a yet-to-be-identified intermediate host(s) may also play a role in the spillover of PRVs from bats to humans. Alternatively, an additional reservoir may play a role in zoonotic transmission of PRV, as is the case with camels and MERS-CoV [30–32].

In this study, we first performed a PRV seroprevalence study on cynomolgus macaques residing in Singapore. We identified 67 out of 517 (12.9%) macaques that were seropositive for PRV, and of these monkeys 34 out of 517 (6.57%) had neutralizing antibodies specifically against PRV3M. This is a significant finding in the context of our recently published study indicating human exposure to PRVs in Singapore [9]. For the first time, we have demonstrated evidence of exposure to PRVs in both human and monkey populations co-residing in the same city/region in close proximity.

We then extended the study to conduct experimental infection of monkeys by PRV to firstly confirm the susceptibility of cynomolgus macaques to PRV infection and secondly to test whether PRV can be transmitted from infected to naïve animals under experimental conditions.

We experimentally infected three seronegative, wild-caught monkeys with PRV3M. We demonstrated that monkeys were susceptible to PRV3M infection, but the infection was subclinical. This confirms the serological surveillance data in relation to susceptibility. The infection displayed no signs and this observation was also not unexpected as it has been reported before that human infection can occur without clinical manifestations [3]. This was in contrast to experimental infection studies in mice, where 100% of infected animals exhibit disease signs and eventually succumb to the infection [5,33]. It is possible that certain risk factors absent in healthy individuals pre-dispose the development of clinical signs in monkeys and humans upon PRV exposure, and further epidemiological studies in patient cohorts would help clarify this.

Furthermore, the demonstration of transmission between animals under experimental conditions supports our hypothesis that mammal(s) other than bats may also play a role in the spillover of PRVs into human population. Wild monkeys are often sighted near human populations in Southeast Asia [34–36], and our data raised the possibility that monkeys in the region may act as an intermediate host for transmission of PRVs to humans and other animals.

While our preliminary data is highly encouraging and significant, further research is needed in better understanding of the bat-monkey interaction and assessing whether other mammals, such as rodents, in the region are also susceptible to PRVs and play a role in the overall transmission landscape.

The infection of monkeys with PRV without observable signs of disease is also important in the context of acting as a potential reservoir host as healthy animals are usually more active with greater area of movement, hence increasing the chance of transmission to other species via contaminated faeces or other routes. Furthermore, as the immunobiology of humans is more closely related to monkeys than bats, host-adaptation within monkeys could serve to enhance the virulence or transmission efficiency of PRV in humans. Given the overlapping habitats of bats and monkeys, interspecies transmission to monkeys and subsequent pathogen adaptation could be a relevant theme in the broader context of bat-borne virus spillover to humans, and warrants further investigation. Finally, the recent report by a group in Thailand on the isolation and characterization of a PRV from monkey faecal samples ruled out contamination of these samples with bat faeces. During analysis of the mitochondrial 12S rRNA gene, bat DNA was not detected in the faecal samples, emphasizing monkeys, especially a long-tailed macaque, as potential reservoirs of zoonotic viruses, such as PRV [18]. We believe that the data obtained from our current study supports the notion that the Thai study represents a first isolation of a PRV replicating in monkeys in this region.

While the current data presented in this study and from previous publications are not sufficient to determine whether non-human primates may act as a reservoir or intermediate host for PRVs, it is important to bear in mind that PRVs have been detected in a wide geographic area and the first PRV was isolated from bats in Australia where non-human primates do not exist.

In summary, our data provides strong support for the hypothesis that non-bat mammals can play a role in the transmission and spillover of PRVs in regions where monkeys and humans co-exist in close proximity. Due to the wide species tropism of PRVs at the cell culture level, we would not be surprised to find other terrestrial mammals as potential hosts of PRVs in the future.
